# Uncertainty analysis of contagion processes based on a functional approach

**DOI:** 10.1038/s41598-023-42041-0

**Published:** 2023-09-19

**Authors:** Dunia López-Pintado, Sara López-Pintado, Iván García-Milán, Zonghui Yao

**Affiliations:** 1 Economics Department, Universidad Pablo de Olavide, 41013 Seville, Spain; 2 Department of Health Sciences, Northeastern University, Boston, 02115-5005 USA; 3 Engineering Department, Universidad de Loyola, 41704 Seville, Spain

**Keywords:** Statistics, Infectious diseases

## Abstract

The spread of a disease, product or idea in a population is often hard to predict. We tend to observe one or few realizations of the contagion process and therefore limited information can be obtained for anticipating future similar events. The stochastic nature of contagion generates unpredictable outcomes throughout the whole course of the dynamics. This might lead to important inaccuracies in the predictions and to the over or under-reaction of policymakers, who tend to anticipate the average behavior. Through an extensive simulation study, we analyze properties of the contagion process, focusing on its unpredictability or uncertainty, and exploiting the functional nature of the data. In particular, we define a novel non-parametric measure of variance based on weighted depth-based central regions. We apply this methodology to the susceptible-infected-susceptible epidemiological model and small-world networks. We find that maximum uncertainty is attained at the *epidemic threshold*. The density of the network and the contagiousness of the process have a strong and complementary effect on the uncertainty of contagion, whereas only a mild effect of the network’s randomness structure is observed.

## Introduction

The ability to predict the spreading behavior of a new idea, product, or disease in a population characterized by a complex network of influences and interactions, either virtual or in person, is a fundamental challenge faced by scientists, such as sociologists, economists and epidemiologists^1–6^. Regardless of whether the focus is on the spread of a new “TV series”, “technology” or “biological virus”, all of these widely different phenomena have in common that they are hard to anticipate^7^. For instance, in the case of an infectious disease, our running example, the rule describing how an agent becomes “infected” by an infectious neighbor is often perceived as stochastic. Infection depends on numerous factors, such as the time and type of exposure or the status of the agent’s immune system. This, together with the complex network of interactions makes the spreading of the disease subject to the accumulation of interdependent and uncertain events which leads to random outcomes. Nevertheless, not all network structures nor contagion rates lead to the same amount of unpredictability. Under some circumstances the past experience (e.g., a disease that has already spread elsewhere) is more useful than in others for anticipating and reacting to further similar events. Thus, understanding the determinants of such inherent uncertainty is of vital importance.

Most of the literature on diffusion in networks has focused on studying the long-run or endemic state of the process, either through simulations or by mean-field theory models that approximate the average behavior^2,6^. Focusing just on average predictions and ignoring the heterogeneity and randomness inherent to most of these processes under appreciates information on the evolution of contagion which, in some cases (e.g., an epidemic), could help avoid mistakes in policy-making. In this paper we analyze the most stylized form of unpredictability, one that simply arises from the stochastic nature of contagion which takes place on a fixed network of interactions. We propose a methodology that helps discerning features of the model that lead to more predictable outcomes by considering the complete time course of contagion. There are recent theoretical studies which significantly contribute to this line of thought by quantifying, in real time, the probability that an epidemic goes supercritical or conversely, dies stochastically. Some of these studies apply the method of probability generating functions^8^, whereas others analyze systems of stochastic differential equations which permit fluctuations in terms of the mean and the variance of infected individuals^9,10^. Advances in this front have been conceived mostly for random networks with arbitrary degree distributions but lacking clustering. In reality, however, the network contains some structure and this could potentially affect the predictions. From an empirical perspective, the recent experience with COVID-19 provides an example of the large variety across infection curves reported throughout the world, which could be due in part to the differences in the underlying network structures of different communities, and also to the inherent unpredictability of the contagion process as will be highlighted in this study^11,12^.

Our work complements previous studies by using a non-parametric functional data approach to analyze, through simulations, how the variability of the contagion process crucially depends on the contagion rate and the properties of the network of interactions. The analysis can be performed on any type of network structure, even those far from random which are hard to analyze theoretically. For concreteness, we focus on the case of the susceptible-infected-susceptible (SIS) epidemiological model^13^ applied to small-world networks^14^, i.e., networks with properties such as high clustering and small average path length, that are quite common in the real world^15^. Alternative scenarios, such as the susceptible-infected-recovered (SIR) epidemiological model, have been analyzed in the Supplementary Information.

### Summary of our findings

The basic unit of study is a random curve (infected proportion curve) defined as the fraction of infected agents in the population as a function of time, where the maximum time period is set exogenous. Multiple realizations of this random process generate a sample of infected proportion curves with characteristics that depend on the given network structure and the diffusion model. We apply robust non-parametric statistical methods based on statistical depth notions for functional data to describe and analyze the properties of the contagion process. In particular, the variability and, ultimately, unpredictability of this process is estimated by a weighted average of the depth-based central regions of the generated sample of infected proportion curves^16,17^. We analyze two types of variance measures; the total (or overall) variance and the before steady state (or short-run) variance. The steady state is defined based on the point-wise median curve, which is smoother than each sampled curve. The infection proportion curve is characterized by a cyclical behavior around the steady state which provokes variability also in the long run. By using the first definition of variance, the total/overall variance, we do not distinguish between variance before or after reaching a steady state which implies that, in the overall variance, the time when the process reaches the steady state is a main component of the measure. In general, those processes that reach the steady state later will have a higher variance. The second measure of variability we propose, the before steady state variance, aims to consider the uncertainty of the process/contagion curve before reaching the steady state, regardless of how long it takes to reach to it, since we are normalizing by this length. Therefore, these two concepts of variability are complementary and jointly provide a more complete information.

We show that the transition from the zero-diffusion to positive (and large)-diffusion regimes occurs abruptly at what has been called the epidemic threshold^5,13,18^, and that this threshold crucially depends on the combination of the contagion rate and the density of the network, but it is generally independent of the structure of the network. Moreover, maximum variability is attained precisely at the epidemic threshold, where the process takes longer time to converge to the endemic state. The overall variance is larger at the threshold than the short-run variance, whereas the opposite is true as the parameters are set further away from the threshold. Finally, the effect of the network randomness is mild (as already mentioned), but we do find that for the lattice or small-world networks the time of convergence to the endemic state is significantly larger than for other network structures (but this does not seem to have important consequences on the other measures analyzed).

## Methodological approach

### The SIS model and small-world networks

The SIS model is typically used to formalize the diffusion of infections that do not confer any long-lasting immunity and thus, upon recovery, individuals become susceptible again (such as the common cold and influenza). There are other related models that can also be conceived for describing social phenomena such as diffusion of innovations, cultural fads, or economic conventions that share the logic of contagion^2,19–21^. The SIR case (Susceptible-Infected-Recovered) is briefly analyzed in the Supplementary Information.

Formally, in the SIS model a susceptible agent may become infected with a probability $\beta$ when interacting with an infectious agent. Reversely, with a probability $\mu$ an infected agent can become susceptible again. For simplicity, we will assume a fixed value of $\mu$ and vary $\beta$. The key parameter is considered as $\lambda =\frac{\beta }{\mu }$, denoted as the “contagion rate” which, when multiplied by the average number of contacts per unit of time coincides with the basic reproductive number $(R_0)$, i.e., the average number of secondary infections caused by a primary case in the random network setting^5,20^. We assume that there is a small initial seed of agents that are spontaneously infected. The infected proportion of agents in the population at certain time *t* is a random function denoted as *X*(*t*). Given the recurrent transition from susceptible to infected and vice-versa, the identities of infected agents vary over time. Also, a realization of the random process, *x*(*t*), is not necessarily monotonic and its particular shape depends on the parameters of the contagion process and on the realization.

**Figure 1:**
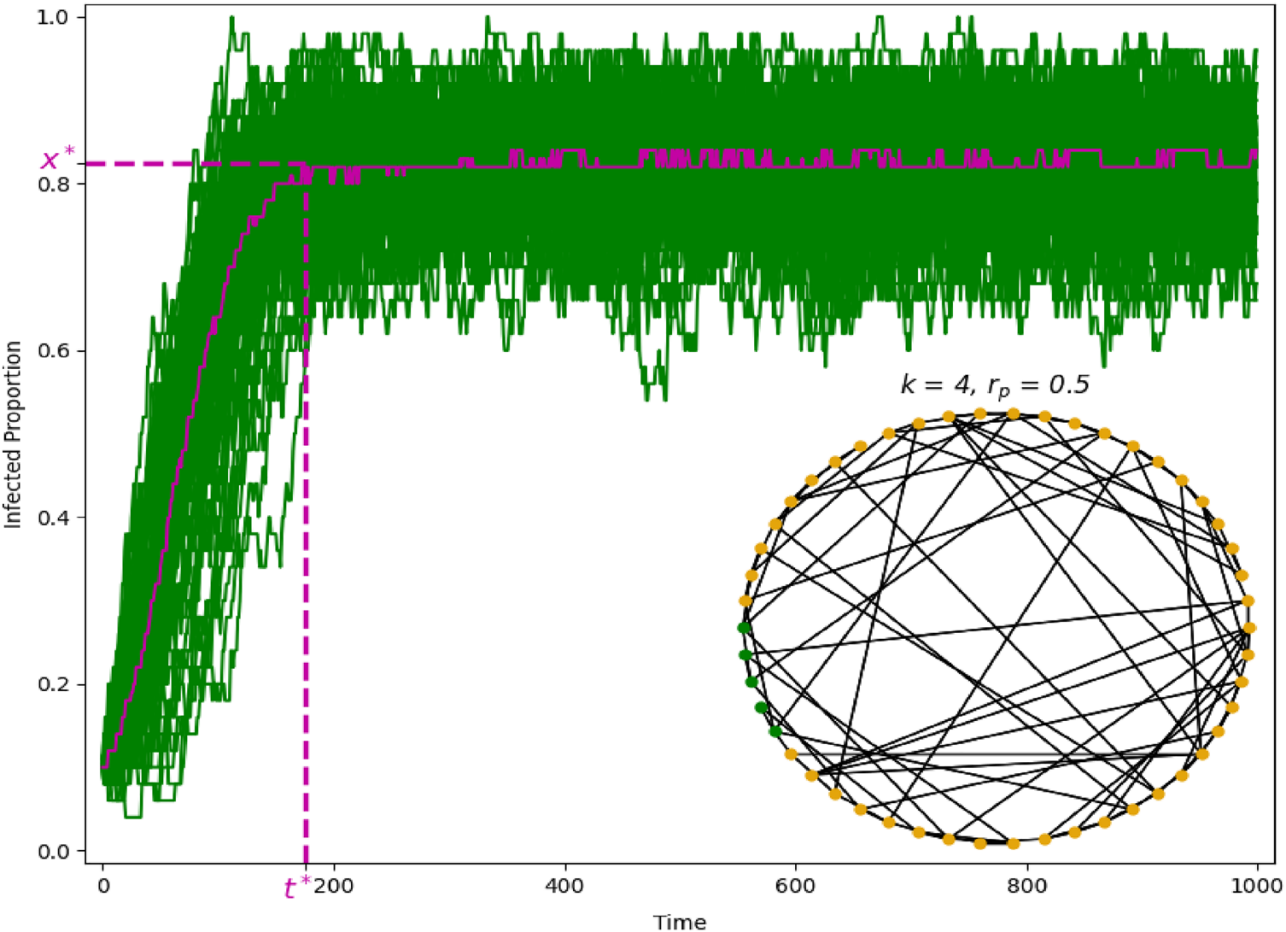
A sample of infected proportion curves. The graph illustrates $n=40$ infected proportion curves simulated given a small-world network and the SIS model with $\lambda =1$. The corresponding network (inset) is formed by $S=50$ nodes, density $k=4$, randomness $r_p=0.5$, and five concentrated initially infected nodes (colored in green). The point-wise median is represented, as well as the estimated steady state point $t^*$ and steady state value $x^*$.

The SIS model is applied to small-world networks^14^. To generate the networks we create a ring over *S* nodes in which each node is connected with its *k* nearest neighbors (or *k* - 1 neighbors if *k* is odd). Each existing link is randomly rewired with a probability $r_p$, which tunes the nature of the network between that of a unidimensional lattice if $r_p$ = 0 and that of a random network if $r_p$ = 1. For small, but positive, values of $r_p$ (as i.e., $r_p=0.01$) we obtain networks satisfying the small-world properties, i.e., high clustering and short average path lengths. A given network will be characterized by its average density (i.e., *k*) and randomness (i.e., $r_p$) as illustrated in the inset of Fig. 1.

### Functional data-based analysis of the contagion process

The SIS model on a fixed network determines a Markov process in which the state of the system at a given time is the profile of nodes that are infected versus those that are susceptible. Due to its stochastic nature, multiple realizations of the process generate different infected proportion curves. Figure 1 shows a sample of 40 infected proportion curves from independent draws of the SIS contagion process with $\lambda =1$, and 5 clustered initially infected nodes (see the graph in the inset of Fig. 1).

#### Steady state point and value

Given a contagion process determined by (*k*, $r_p$) and $\lambda$, let *X* be a random function, as defined before, where *X*(*t*) is the infected proportion of individuals at time *t*, with $t\in [0,T]$, *T* being the maximum time considered and $X(t)\in [0,1]$. Let $x_1, x_2,\ldots ,x_n$ be a sample of *n* independent realizations from the random function *X*, i.e., a sample of infected proportion curves. We denote as *M*(*t*) the point-wise median curve, that is,

$$\begin{aligned} M(t)=median\,\left(x_1(t), x_2(t),\ldots ,x_n(t)\right). \end{aligned}$$

The “steady state point” (*SSP*), denoted by $t^*$, is the time period where *M*(*t*) starts converging to an equilibrium and slightly fluctuates around this value, i.e., when the point-wise median reaches “approximately” a stationary state. In fact, the *SSP* is interpreted as the moment in time that separates the short-run versus long-run of the process and we calculate it by taking the first derivative of the point-wise median curve (numerical difference approximation) and smoothing it using a moving average approach. This is illustrated in Fig. S1 in the Supplementary Information. The *SSP* is defined as the point in time for which such estimated derivative function is close to zero, e.g., reaches a value lower than a predefined small threshold, from such point on-wards. Figure 1 illustrates the corresponding point-wise median given a sample of infected proportion curves and its *SSP*. The “steady state value” (*SSV*), denoted by $x^*$, is computed as the average of the values taken by *M*(*t*) after the steady state point is reached (see $x^*$ in Fig. 1). In the simulation study the maximum time *T* is set high enough so that $t^*$ is always smaller than *T*. Formally,

$$\begin{aligned} SSV \equiv x^*=\frac{\int _{t^*<t<T} M(t) \quad dt}{T-t^*}. \end{aligned}$$

As shown in Fig. 1 the curves in the sample fluctuate around the steady state value *SSV*, defined based on the point-wise median. This is the long-run (or after steady state) variance inherent in the finite version of the SIS model. This type of variance, which becomes smaller as the size of the network becomes larger, is quite different to the one we want to focus on in the paper. This leads to two definitions of variance, introduced in the next section, both of them based on the idea of functional data depth.

#### A novel depth-based measure of variance

There are many different ways of measuring the dispersion or variability in a sample of curves. We calculate the variability of a sample of curves in a robust and non-parametric fashion by using the notion of functional data depth. The idea of data depth was originally proposed to rank multivariate observations from center outward^22–24^ and it was later extended to functional data. It is a powerful exploratory tool for analyzing the distribution of samples of curves^16,25,26^. Functional data depth provides a rigorous way of ranking functions in terms of their representativeness/centrality with respect to the sample and of defining non-parametric and robust functional statistics. The higher the depth of a function within a sample the most representative/central it is, in contrast, low depth implies that the observation is in the outer-skirt of the sample distribution and it is a potential outlier. We use the “modified band depth” (*MBD*) concept, one of the first definitions of functional depth proposed in the literature, which is based on the regions/bands created by all possible pairs of curves in the sample^16^.

Let $x_1, \ldots , x_n$ be a sample of curves that are independent realizations of the random function *X* defined on the interval [0, *T*] and taking real values in the interval [0, 1]. The (sample) *MBD* of a function *x* with respect to the sample $x_1, \ldots , x_n$, is defined as follows:

$$\begin{aligned} MBD(x)= \begin{pmatrix} n\\ 2 \end{pmatrix}^{-1}\sum _{1\le i_1 < i_2 \le n} \Lambda \{B(x;x_{i_1}, x_{i_2} )\}, \end{aligned}$$

where

$$\begin{aligned} B(x;x_{i_1}, x_{i_2} ) =\{t \in [0,T]: \min _{r=i_1,i_2} x_r(t)\le x(t) \le \max _{r=i_1,i_2} x_r(t)\}, \end{aligned}$$

and $\Lambda$ is the Lebesgue measure on the real line normalized by *T*. Hence, *MBD*(*x*) measures the proportion of time the function *x* is in the band determined by $x_{i_{1}},x_{i_{2}}$, averaged over all possible bands defined by pairs of functions from the sample. Note that there is a population version of this sample depth that we do not use in this paper^16^, so we have avoided a notation distinguishing between the population and sample depths, i.e., *MBD* verse $MBD_n$, as we are always considering the later.

**Figure 2:**
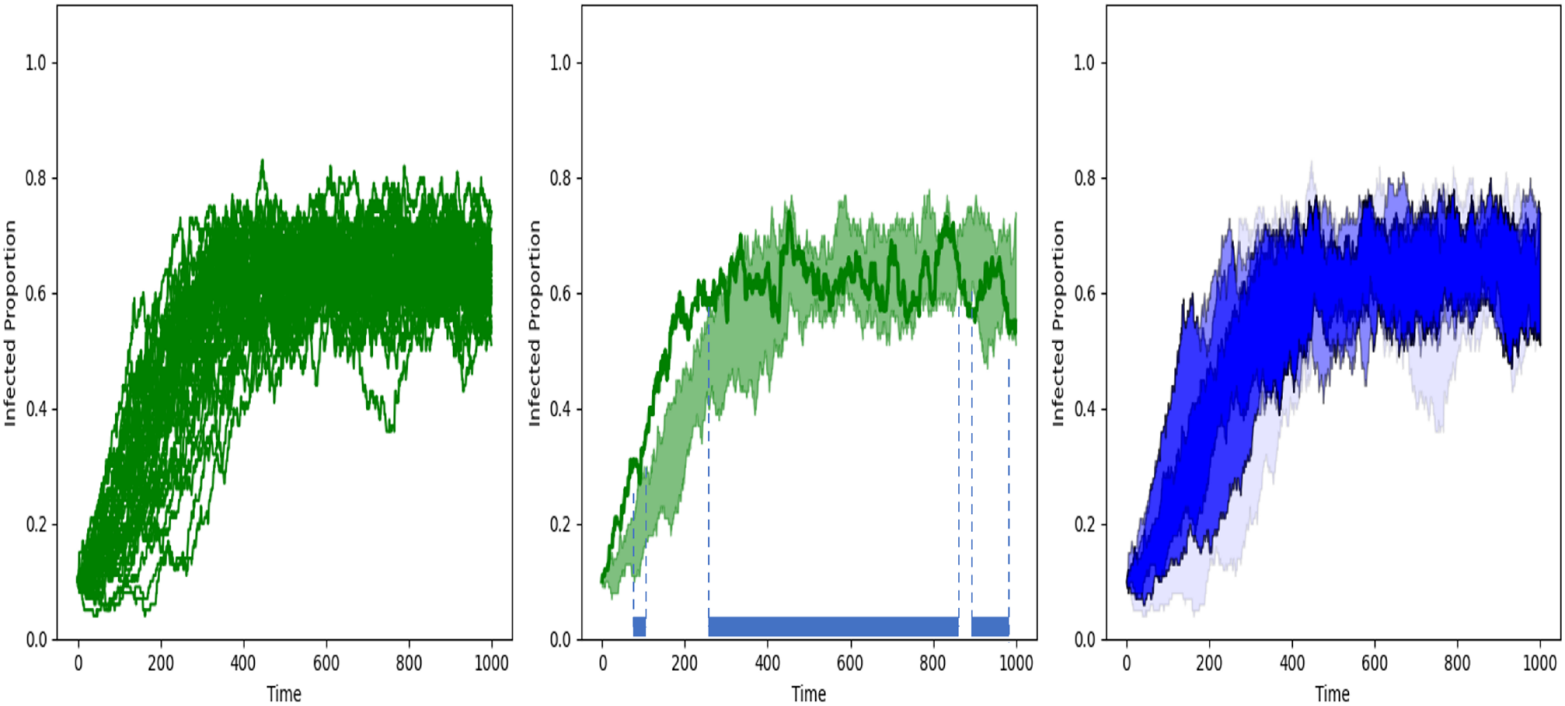
Left. Sample of curves. Middle. Visualization of how the modified band depth is calculated for the bold curve as the proportion of time such curve is in the band determined by two curves from the sample; the average of these proportions over all possible pairs of curves from the sample is the modified band depth. Right. Blue gradient representing the regions determined by the 25%, 50%, 75% and 100% deepest curves in the sample based on the modified band depth (*MBD*). See text for details.

In Fig. 2 we illustrate how to calculate *MBD* of a sample of infected proportion curves (left graph) by representing the band generated by two random curves from the sample and the proportion of time that a third curve from the sample, say *x* (bold green), is inside the band (as illustrated in the middle graph). *MBD*(*x*) considers this “in band time proportion” of *x*, averaged over all possible pairs of curves from the sample and it assigns to the curve *x* a number between zero and one. The higher this number the more representative the curve *x* is within the sample. *MBD* then generates a reasonable and rigorous ranking for the sample of curves from the deepest or more central curve to the least deep or extreme one^16^. Based on the *MBD* values one can define robust location estimators, such as the median or trimmed mean functions. For example, the median curve is defined as the curve from the sample with highest *MBD* value and the 0.50-trimmed mean is the average of the 50% deepest curves from the sample.

The *MBD* ranking can also be used for measuring the variance or dispersion of a sample of curves in a robust and non-parametric way. In particular, the “area of the *p* central region” ($ACR_p$) defined formally below determines the variance of a sample of curves as the area of the region encompassed exclusively by the proportion *p* of deepest curves from the sample, where $p \in [0,1]$. This idea of measuring dispersion based on data depth rankings was first introduced for multivariate data^27^ and later extended to functional data^16,28^.

More concretely, $ACR_p$ based on *MBD* is defined as:

$$\begin{aligned} ACR_p=\frac{\int _0^T (\max _{j=1,\ldots \lceil n\cdot p\rceil } x_{[j]}(t)-\min _{j=1,\ldots \lceil n \cdot p\rceil } x_{[j]}(t) )\quad dt}{T} ,\end{aligned}$$

where $x_{[1]},\ldots , x_{[n]}$ are the center-outward ranked curves, with $x_{[1]}$ being the deepest (most central or median) curve, $x_{[n]}$ being the most outlying curve, and $\lceil n \cdot p\rceil$ rounding up to the nearest integer. The advantage of this measure of variance is that it is intuitive, non-parametric, easy to calculate, and robust, as it neglects the possible outliers in the sample. It is straightforward to show that these areas are nested, that is, if $p\le {\tilde{p}}$ then $ACR_{p}\le ACR_{\tilde{p}}$. Also, $ACR_{0.50}$ is the area of the central region/band determined by the 50% deepest curves from the sample and it can be seen as an extension of the standard univariate Interquartile Range (*IQR*) concept to functional data^16^. This notion of variance out-performs alternative definitions of dispersion for functional data, especially in the presence of outliers^28^.

On the other hand, an issue with the standard $ACR_{0.50}$ concept is that considering the 50% deepest curves (or *p*=0.50) instead of the 75% or 25% is quite arbitrary. Moreover, by concentrating only on the deepest subset of curves part of the information obtained in the sample and its ranking is lost. We thus propose a novel measure of variance that weights the more central curves more than the less central ones, while still including all curves in the sample. A natural way to formalize this idea is to consider a “weighted average” of $ACR_p$ for different values of *p*. Precisely, given the distribution of *MBD* values obtained from the sample, the *q*-quantiles divide such sample in *q* groups of same frequency, according to *MBD* (i.e., tertiles if *q*=3, quartiles if *q*=4, and so on, and so forth). We define the “*q*-weighted average of the central region area” ($WACR_q$) as follows:

$$\begin{aligned} WACR_q=\sum _{p \in \{\frac{1}{q},\frac{2}{q},\ldots ,\frac{q-1}{q}, 1\} } \alpha _{p,q} \cdot ACR_p, \end{aligned}$$

where

$$\begin{aligned} \alpha _{p,q} = \frac{\displaystyle \sum _{i=\lceil n \cdot (p-(1/q))+1 \rceil }^{ \lceil n\cdot p \rceil } MBD_{[i]}}{\displaystyle \sum _{i=1}^n MBD_{[i]}}, \end{aligned}$$

and $MBD_{[1]}\cdots MBD_{[n]},$ represent the *MBD* values of the sample curves, from the deepest to the least deepest one. In other words, $MBD_{[i]}=MBD(x_{[i]})$ for $i=1,\ldots ,n$. Note that *q* represents the numbers of groups we divide the sample in, and is set to be a whole number between 1 and n. For example, given *q* greater than one, then, if $p=2/q$ in the definition of $WACR_q$ the term $ACR_p$ is weighted by $\alpha _{p,q}$ which corresponds with the sum of the *MBD* indexes of all curves in the second *q*-quantile divided by the sum of all *MBD* indexes. Since the *MBD* are ordered from highest to lowest, the second *q*-quantile corresponds to the second group of curves with highest depths. Also, by definition, these weights, $\alpha _{p,q}$, add up to one. Note that the dispersion of the deepest curves will be overweighted in the final $WACR_q$ dispersion value, but all curves in the sample are considered. In the case of $q=1$, ${WACR_1}$ is just the normalized area determined by the whole sample of curves. Following the notation introduced above, let us also define the “relative *MBD*” of the curve $x_{[i]}$ for $i=1,\ldots ,n$ as

$$\begin{aligned} RMBD_{[i]} =\frac{\displaystyle MBD_{[i]}}{\displaystyle \sum _{j=1}^n MBD_{[j]}}, \end{aligned}$$

which are indexes defined in the interval [0,1], decreasing with respect to *i* and that add up to 1. Some basic properties of ${WACR_q}$ are established below:

##### Proposition 1

The ${WACR_q}$ measure satisfies the following simple properties:

(i) $$WACR_q\in [0,1]$$ for any $$q \in \{1,2,...,n\}$$.

(ii) $WACR_q\le WACR_1$ for any $q \in \{1,2,...,n\}$.

(iii) Given two samples of curves $\tilde{x}=\{x_1, \ldots , x_n\}$ and $\tilde{y}=\{y_1, \ldots , y_n\}$ satisfying the following conditions:

(a) $\sum _{i=1}^k{RMBD}{^{\tilde{y}}}_{[i]}\le \sum _{i=1}^k{RMBD}{^{\tilde{x}}}_{[i]}$ for any $k \in \{1,2,...,n\}$, and

(b) $$ACR_p(\tilde{x})\le ACR_p(\tilde{y})$$ for all $$p \in [0,1]$$,

then for any $q \in \{1,2,...,n\}$

$$\begin{aligned} WACR_q(\tilde{x}) \le WACR_q(\tilde{y}), \end{aligned}$$

where ${RMBD}{^{\tilde{z}}}_{[i]}$, $ACR_p(\tilde{z})$ and $WACR_q(\tilde{z})$ are interpreted as the corresponding measures evaluated for any given sample $\tilde{z}$.

The proof of this proposition is the following: Property (i) indicates that our measure of unpredictability (or variance) is a positive number between 0 and 1 which is a straightforward consequence of $ACR_p\in [0,1]$, $\sum _{p \in \{\frac{1}{q},\frac{2}{q},\ldots ,\frac{q-1}{q}, 1\}} \alpha _{p,q}$ =1 and $\alpha _{p,q}\in [0,1]$ for all $p \in [0,1]$ and $q \in \{1,2,\ldots ,n\}$. Property (ii) holds as a consequence of $ACR_{p}\le WACR_1$=$ACR_{1}$ for all $p \in [0,1]$ by the monotonicity of $ACR_{p}$ in *p* and the fact that $WACR_q$ is (by definition) a weighted average of *q* values of $ACR_{p}$. Nonetheless, this does not imply that the values $WACR_q$ are decreasing with respect to *q*. In fact, there are many parameter specifications for which we observe that although $WACR_q$ has a decreasing trend it is indeed non-monotonic (see Fig. S2). Finally, property (iii) provides a sufficient condition for ordering two sample of infected proportion curves in terms of our proposed measure of variance ($WACR_q$). The proof of this property is considerably more challenging. First, let us show that if, for any given sample $\tilde{z}$, we view $\alpha {^{\tilde{z}}}_{p,q}$ as a probability distribution with respect to *p*, then it is the case that, given condition (a), $\alpha {^{\tilde{y}}}_{p,q}$ first order stochastic dominates $\alpha {^{\tilde{x}}}_{p,q}$. This is due to the fact that for any $k \in \{1,2,\ldots ,q\}$

$$\begin{aligned} \sum _{p \in \{\frac{1}{q},\frac{2}{q},\ldots ,\frac{k}{q}\} }\alpha _{p,q}=\sum _{i=1}^k{RMBD}_{[i]}, \end{aligned}$$

and thus, following condition (a),

$$\begin{aligned} \sum _{p \in \{\frac{1}{q},\frac{2}{q},\ldots ,\frac{k}{q}\} }\alpha {^{\tilde{y}}}_{p,q}\le \sum _{p \in \{\frac{1}{q},\frac{2}{q},\ldots ,\frac{k}{q}\} }\alpha {^{\tilde{x}}}_{p,q}, \end{aligned}$$

which indeed shows that $\alpha {^{\tilde{y}}}_{p,q}$ first order stochastic dominates $\alpha {^{\tilde{x}}}_{p,q}$. Since, by definition, $ACR_{p}$ is an increasing function of *p* then, using a property of first order stochastic dominance, it holds that:

$$\begin{aligned} WACR_q(\tilde{x})\equiv \sum _{p \in \{\frac{1}{q},\frac{2}{q},\ldots ,\frac{q-1}{q}, 1\} } \alpha {^{\tilde{x}}}_{p,q} \cdot ACR_p{(\tilde{x})}\le \sum _{p \in \{\frac{1}{q},\frac{2}{q},\ldots ,\frac{q-1}{q}, 1\} }\alpha {^{\tilde{y}}}_{p,q} \cdot ACR_p{(\tilde{x})}. \end{aligned}$$

To complete the proof note that, given condition (b), it is true that:

$$\begin{aligned} \sum _{p \in \{\frac{1}{q},\frac{2}{q},\ldots ,\frac{q-1}{q}, 1\} }\alpha {^{\tilde{y}}}_{p,q} \cdot ACR_p{(\tilde{x})}\le \sum _{p \in \{\frac{1}{q},\frac{2}{q},\ldots ,\frac{q-1}{q}, 1\} }\alpha {^{\tilde{y}}}_{p,q} \cdot ACR_p{(\tilde{y})}\equiv WACR_q(\tilde{y}), \end{aligned}$$

which in turn implies that,

$$\begin{aligned} WACR_q(\tilde{x})\le WACR_q(\tilde{y}), \end{aligned}$$

and this completes the proof.

The value given by $WACR_q$ can be interpreted as the total or overall variance of the process defined over an exogenous and large time interval *T*. This value combines the short-run and long-run variance. The short-run variance evaluates the process in the run-up to the steady state, whereas the long-run variance measures the variance at the point-wise median’s steady state. A disadvantage of the total variance measure defined above is that it depends on *T* and the larger the *T*, the more weight is given to the variance in the long-run. However, in many real processes external interventions modify the properties of the network or contagion rate before the long-run is even reached and thus, the behavior in the long-run is not often materialized. For these reasons, we also consider an alternative measure of variance which builds on the definition of $WACR_q$ but truncates it at the time period $t^*$. This short-run variance will be referred to as the “*q*-before weighted average of the central region” ($BWACR_q$) and equals:

$$\begin{aligned} BWACR_q=\sum _{p \in \{\frac{1}{q},\frac{2}{q},\ldots ,\frac{q-1}{q}, 1\} } \alpha _{p,q} \cdot BACR_p, \end{aligned}$$

where the “before average central region” for any given $p \in [0,1]$ is defined as

$$\begin{aligned} BACR_p=\frac{\int _0^{t^*} (\max _{j=1,\ldots \lceil n\cdot p\rceil } x_{[j]}(t)-\min _{j=1,\ldots \lceil n \cdot p\rceil } x_{[j]}(t) )\quad dt}{t^*} \end{aligned}$$

It is straightforward to see that Proposition 1 also holds for $BWACR_q$.

In the simulation study presented in the next section time is discretized and thus, the integrals used to define *SSV*, $ACR_p$ and $BACR_p$ are substituted by sums. We analyze both the total and short-run variance concentrating on the case $q=4$. In other words, we assume the *MBD* values are divided into quartiles (four groups) from the highest to the lowest values and calculate $WACR_4$ and $BWACR_4$, i.e., the weighted average of the *p*-central regions $ACR_p$, or $BACR_p$, respectively, for $p \in \{0.25,0.5,0.75,1\}$. Figure 2 (right graph) represents, for a sample of curves and in a blue color gradient, the area of the nested central regions, where the darker the colour the deeper the curves are. Recall that, for example, if $p=0.75$ the value of $ACR_{0.75}$ in the definition of $WACR_4$ is weighted by the sum of the relative *MBD* indexes of the curves included in the third quartile of the *MBD* distribution. Therefore the extreme or outlier curves are under-weighted in the proposed measure of variance. For simplicity, in what follows we will use the notation *WACR* (*BWACR*) for the measure of total variance (short-run variance) and avoid subscript 4.

## Simulation results

The family of small-world networks considered for the simulations are formed by $S=1000$ nodes, $10\%$ of which are infected initially in a (randomly selected) “neighborhood” of the network. We consider a clustered (concentrated) seed because this is a reasonable assumption for many real-world examples of diffusion (e.g., the appearance of a disease that mutates from an animal to a human virus in a certain location). In particular, a random node in the network is infected initially as well as its neighbors, neighbors of neighbors, etc, until 10% of these nodes are infected in the first period. We have also considered the case with a 1% initial seed. Most findings coincide for a smaller initial seed, although there are some differences that will be pointed out later in the text (see the Supplementary Information for details). The simulation study assumes average degrees *k*, ranging from 4 to 14 (only pair values), rewiring probabilities $r_p$, ranging from 0 to 1 and contagion rates $\lambda$ taking values from 0.1 to 2.1. For every network created with parameters (*k*, $r_p$), a contagion rate $\lambda$, and a fixed initial seed, we run 100 repetitions of the SIS dynamics and derive a sample of infected proportion curves. We also set $T=9000$ which guarantees that the process reaches the steady state for all the parameter configurations considered. We summarize and visualize the results in a schematic way by representing, under the different parameter specifications considered, the *SSP*, *SSV*, *WACR* and *BWACR* measures as defined above.

### The density of the network and the contagion rate

**Figure 3:**
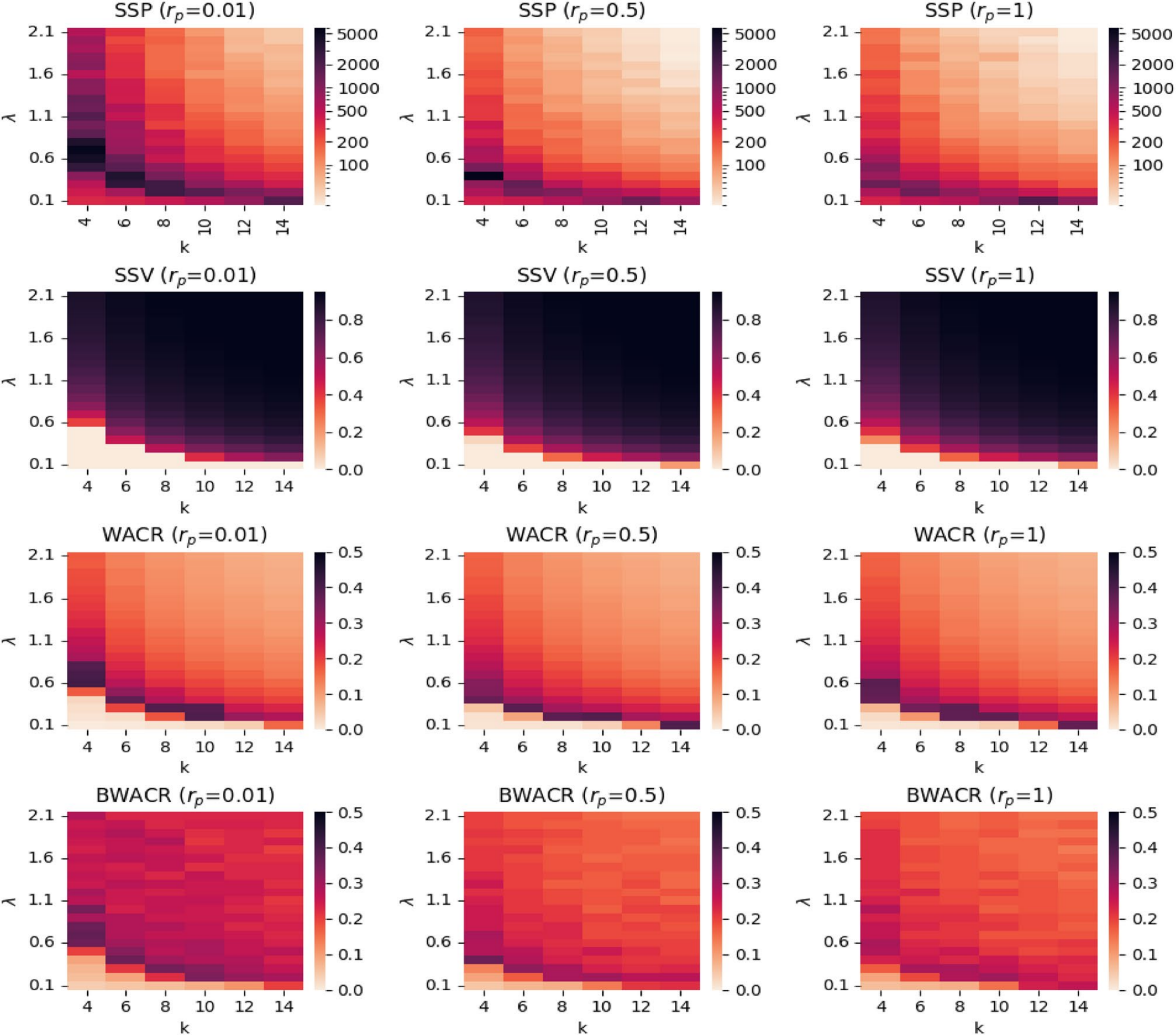
Heatmaps of the steady state values and unpredictability. *SSP*, *SSV*, *WACR* and *BWACR* values (top, middle and bottom rows, respectively) as a function of $\lambda$ and *k* at three levels of $r_p$ (left, middle and right columns, respectively). The darker the colour the higher the values. The *SSP* values are represented in logarithmic scale.

We focus here on the joint effect on the diffusion process of the density of the network and the contagion rate (i.e., *k* and $\lambda$). The results are summarized in Fig. 3, where we show, through the intensity of the colours in a heatmap, how the *SSP*, *SSV*, *WACR* and *BWACR* values (first, second, third, and fourth rows, respectively) depend on *k* and $\lambda$, for three levels of the rewiring probability such as $r_p$=0.01, 0.5 and 1 (left, middle and right columns, respectively). The case $r_p$=0.01 is of particular interest because it corresponds with a network structure satisfying the small-world network properties (high clustering and low average path lengths) which are common in real networks.

Comparison of the plots (left, middle and right) in every row indicates that the contagion patterns for the three network structures are quite similar, thus, the description of the findings can be done jointly. There are two clearly distinct regimes regarding diffusion; zero-diffusion and positive-diffusion (see the white versus red regions in the graphs represented in the second row of Fig. 3). The parameter configurations for which this, rather abrupt, transition occurs corresponds with the theoretical concept of an epidemic threshold of $\lambda$, denoted by $\lambda ^*$, a well-known phenomenon for the SIS model. In the simulations we will consider the epidemic threshold with respect to $\lambda$ as the first value of $\lambda$ for which *SSV* is positive (given *k* and $r_p$). Similarly we can define the epidemic threshold with respect to *k* as the first value of *k* for which *SSV* is positive (given $\lambda$ and $r_p$), and analogously with respect to $r_p$, given the other two parameters. In some cases the concept of epidemic threshold does not apply. For example, for $\lambda$ sufficiently high (e.g., $\lambda$ above 0.6 in the graphs in the second row) there is positive diffusion for all values of *k* considered in the simulations, and therefore, the epidemic threshold with respect to *k* does not apply with our parameter specifications. Our main finding is that the variance (short-run and overall) of the contagion process is maximized at the epidemic threshold; it has an increasing trend below the threshold and a decreasing trend above it. This is true, largely because the time it takes to the steady state reaches its peak/maximum at this threshold, as illustrated in the first row of Fig. 3. However, note that the short-run variance (*BWACR*), which is normalized by $t^*$, is also higher at the epidemic threshold, although only slightly (see the fourth row in Fig. 3).

Moreover, the set of level curves for *SSV* as a function of *k* and $\lambda$ have a decreasing and convex pattern (i.e., a decreasing hyperbolic shape) which means that, apart from substitutes, there exist some degree of complementarity between the contagion rate and the density of the network regarding the diffusion levels. This implies that the combination of moderate network density with a moderate contagion rate enhance diffusion, in contrast to more extreme values of each parameter (as e.g., high value of *k* but low $\lambda$, or vice-versa) and this pattern is true for all network structures considered. Note that *SSP* and the variability measures *WACR* and *BWACR* also have a similar pattern with level curves that have roughly a decreasing hyperbolic shape.

**Figure 4:**
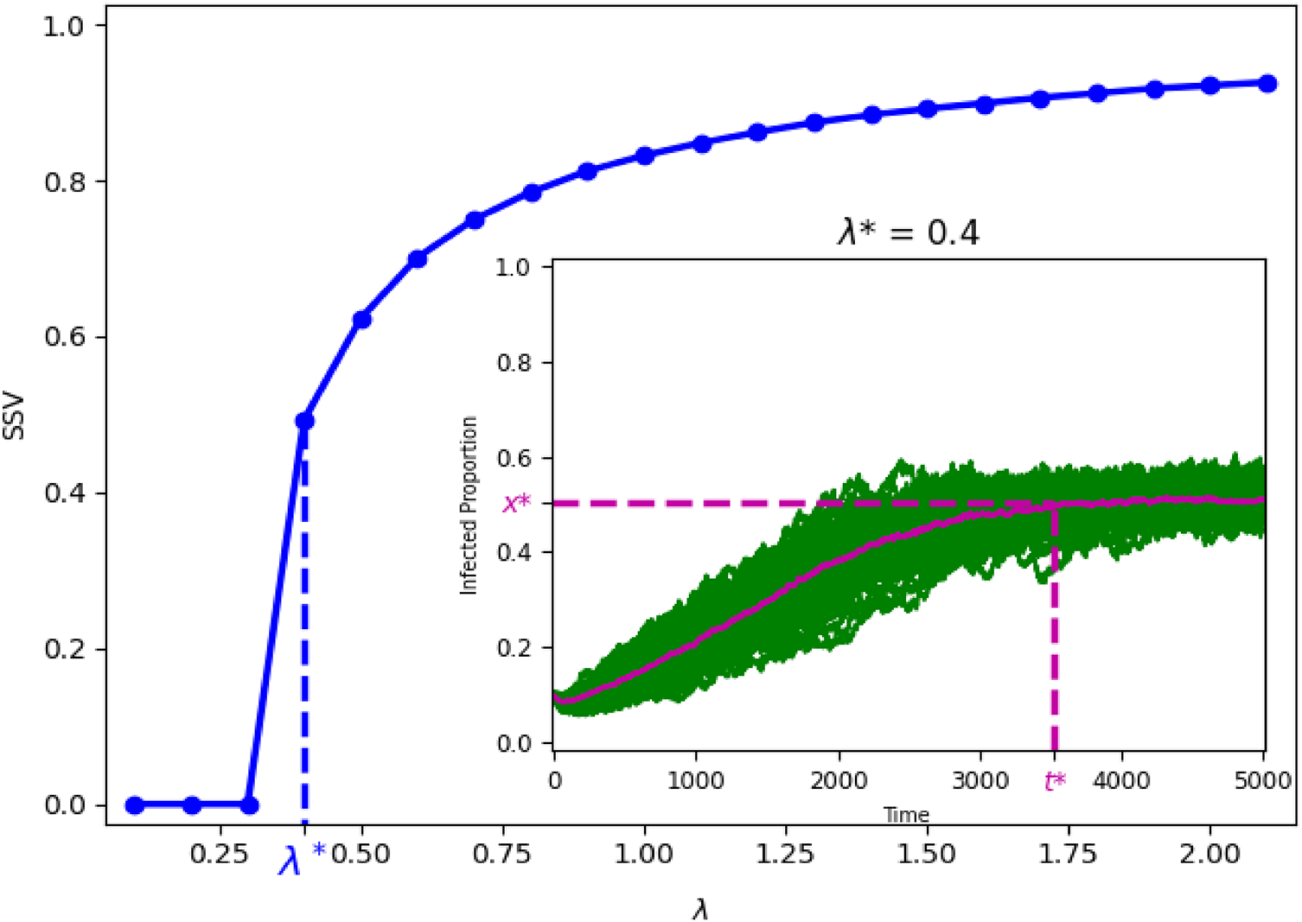
Representation of *SSV* as a function of $\lambda$ in the case *k*=8 and $r_p$=0.01. The value $\lambda ^*$ corresponds with the epidemic threshold and the situation of maximum uncertainty (regarding *WACR* and *BWACR*). The sample of proportion infected curves at $\lambda ^*$ is shown in the inset of the figure.

**Figure 5:**
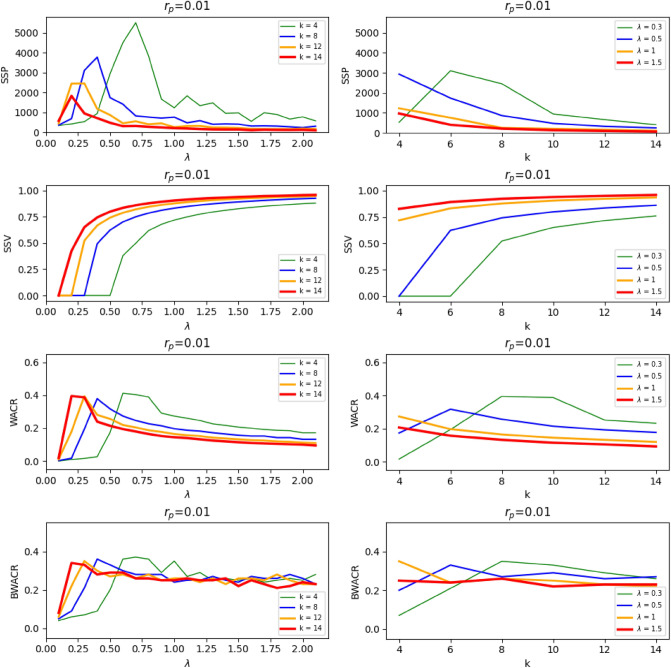
Left column. Representation of *SSP*, *SSV*, *WACR* and *BWACR* as a function of $\lambda$ for *k*=4, 8, 12, and 14, and $r_p$=0.01. Right column. Representation of *SSP*, *SSV*, *WACR* and *BWACR* as a function of *k* for $\lambda$=0.3, 0.5, 1, and 1.5, and $r_p$=0.01.

To analyze these findings in further detail, we focus on the contagion rate as the explanatory variable and illustrate, in Fig. 4, the steady state value as a function of $\lambda$ for a given network (in this case, $k=8$ and $r_p$=0.01), with increments of 0.1 in $\lambda$. In the inset graph of Fig. 4 we show the simulated sample of 100 infected proportion curves for $\lambda ^*$=0.4, which is the epidemic threshold value of $\lambda$ in this setting. In Fig. 5 (left column) we extend the analysis by representing the measures *SSP*, *SSV*, *WACR* and *BWACR* as functions of $\lambda$ for the cases $r_p$=0.01 and *k*=4, 8, 12 and 14. We confirm that *SSP*, *WACR* and *BWACR* curves are maximized at the same value of $\lambda$, which coincides with the epidemic threshold as shown in the *SSV* graphs. In Fig. 5 (right column) we perform an analogous study considering now the density of the network as the explanatory variable represented in the *x* axis. In particular, *SSP*, *SSV*, *WACR* and *BACR* are represented as functions of *k* for the cases $r_p$=0.01 and $\lambda$=0.3, 0.5, 1 and 1.5. The general features coincide with the previous analysis. For instance, variance reaches a peak in the epidemic threshold, whenever such threshold exists (i.e., for $\lambda$=0.3 and 0.5). We find, however, that *SSP* is not maximized at the epidemic threshold, but at a lower value of *k* for $\lambda$=0.5. For the cases where there is no epidemic threshold since the *SSV* is always positive (for $\lambda$=1 and 1.5), *SSP* and *WACR* tend to decrease with *k*, however *SSV* is stable or slightly increasing.

### The randomness of the network and the contagion rate

**Figure 6:**
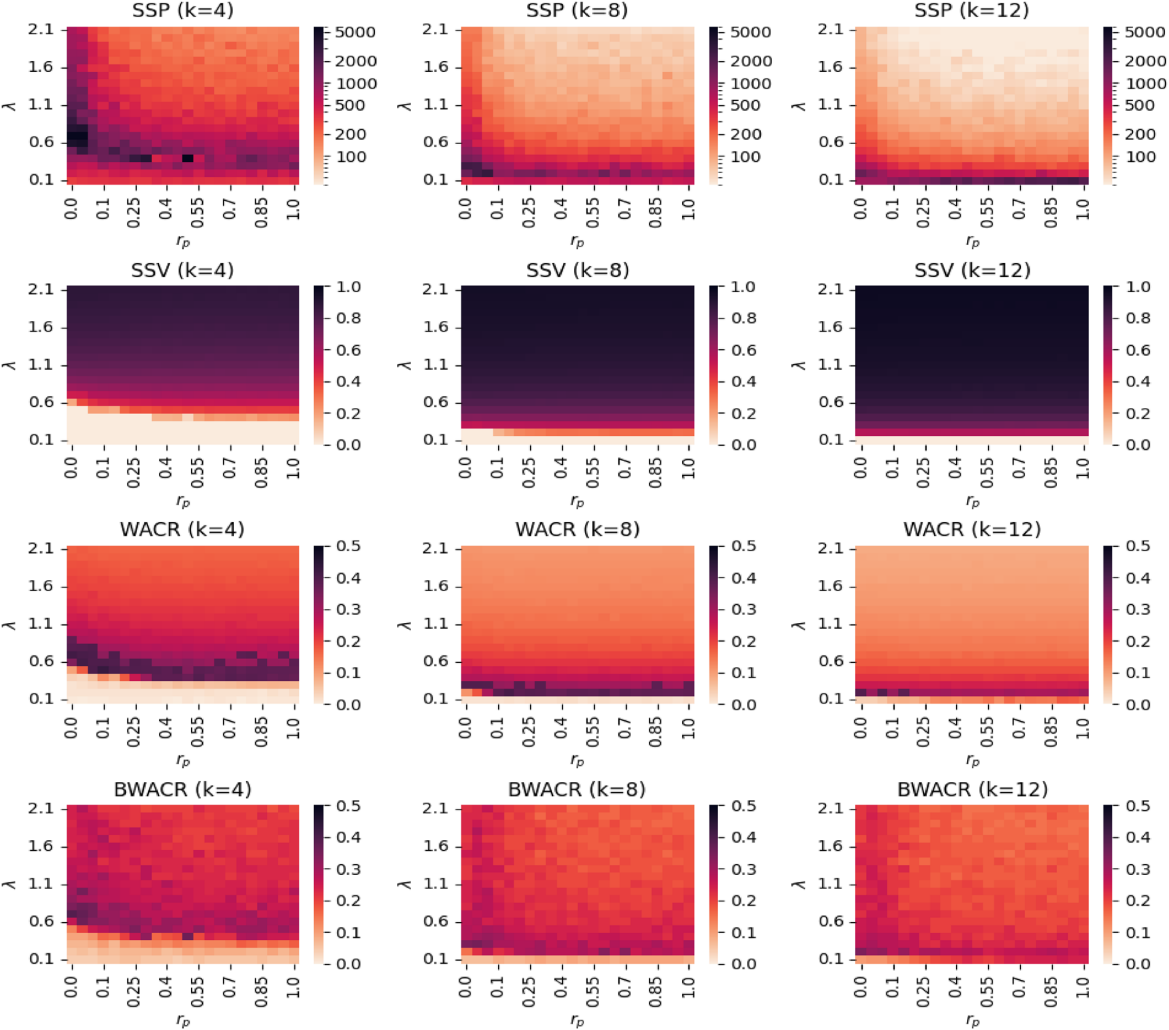
Heatmaps of the steady state and unpredictability. *SSP*, *SSV*, *WACR* and *BWACR* values (top, middle and bottom rows, respectively) as a function of $\lambda$ and  $r_p$ at three levels of *k* (left, middle and right columns, respectively). The darker the colour the higher the values. The *SSP* values are represented in logarithmic scale.

**Figure 7:**
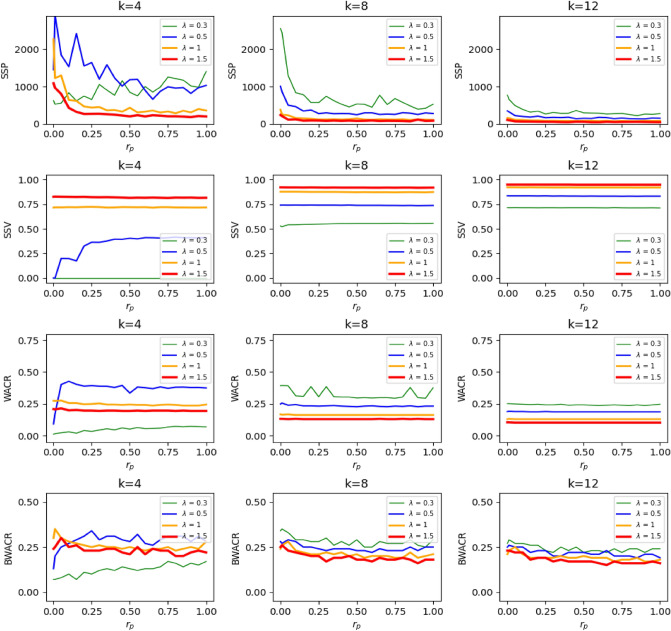
Uncertainty and network randomness. *SSP*, *SSV*, *WACR* and *BWACR* values as a function of $r_p$ given four values of $\lambda$ and *k* = 4, 8 and 12 (left, middle and right columns, respectively).

The results shown in Fig. 3 suggest that the effects of the network randomness (or structure) on the contagion process are minor (by comparing first, second and third columns). To confirm this, we illustrate in Fig. 6 the joint effect on diffusion of the rewiring probability ($r_p$) and the contagion rate ($\lambda$), and compute the measures *SSP*, *SSV*, *WACR* and *BWACR* as before. We test the process at three different levels of *k* such as $k=4$, $k=8$ and $k=12$ (left, middle, and right columns, respectively, in Fig. 6). It is well-known that as $r_p$ increases, clustering decreases in the network and so does the average path length, although the average degree (or density) of the network remains constant^14^. Moreover, the degree distribution becomes more heterogeneous, converging to a Poisson distribution for the random network case (i.e., $r_p$=1) when the size of the network is sufficiently large^29^. We find that the effects of $r_p$ on the four measures analyzed (i.e., *SSP*, *SSV*, *WACR* and *BWACR*) are mild, (especially compared to $\lambda$’s and *k*’s effects). In order to formalize this idea, recall that the epidemic threshold with respect to $r_p$ is defined as the first value of $r_p$ for which *SSV* is positive (given $\lambda$ and *k*), thus, separating the zero-diffusion regime from the positive-diffusion regime. As observed in Fig. 6 (second row), this threshold rarely exists as diffusion is positive or not, usually independently of $r_p$. In other words, $r_p$ typically plays no significant role on diffusion. For instance, in the first column, second row of Fig. 6 we observe that there exists an epidemic threshold with respect to $r_p$ only for the cases $\lambda$=0.4 and 0.5. For values of $\lambda \le 0.3$ there is zero diffusion regardless of $r_p$, whereas if $\lambda \ge 0.6$ there is positive diffusion for every value of $r_p$. Moreover, the actual value of *SSV* (and not only whether it is positive or not) does not seem to depend much on $r_p$ neither. We do find that for a wide range of values of $\lambda$ and small density networks (e.g., *k*=4 or *k*=8) the time of convergence to the steady state decreases with network randomness, but this effect disappears once $r_p$ is sufficiently high. This effect might be a consequence of the degree distribution becoming more heterogeneous as $r_p$ increases, which is known to enhance diffusion in the SIS model (for purely random networks)^13^. Also, the uncertainty of the process does not seem to depend much on $r_p$ and it has a somewhat irregular behavior in the short-run. To properly discuss these findings we represent the measures *SSP*, *SSV*, *WACR* and *BWACR* as functions of $r_p$ for different selected values of *k* and $\lambda$ (see Fig. 7). We confirm that the effect of $r_p$ is minor. For instance, in the case of “high” density (second and third columns of Fig. 7), *SSV* is always positive and roughly constant with respect to $r_p$ for all values of $\lambda$ considered. The time of convergence is decreasing with respect to $r_p$ for small values of $r_p$ and constant otherwise. Furthermore, the short-run variance has a slightly decreasing trend, whereas the overall variance is approximately constant. For the case of a small density network (first column of Fig. 7 for which *k* = 4), and regarding *SSP* and *WACR*, for a low value of $\lambda$ (i.e., $\lambda$ = 0.3), the trend is increasing, but for higher values of $\lambda$ (i.e., $\lambda$ = 0.5, 1 or 1.5) the trend is decreasing. The reason being that if $\lambda$ is low, there is some diffusion only when the network is sufficiently random, which is when some uncertainty might arise, since, otherwise, the disease simply disappears soon. If $\lambda$ is higher, diffusion takes place for all network structures considered, but it appears that it does so in a more irregular and uncertain fashion in the regular network case than in the random network case, although the magnitude of the effect is minor. To summarize, the structure of the network, given its density, has only minor effects on the uncertainty properties of the contagion process.

### Further results

#### Changes in the seed

In this section we analyze the robustness of our results regarding the choice of the initial seed. A disease or idea typically originates in a small cluster of agents which is why we replicated the analysis but with a 1% cluster of initial infected agents, instead of a 10% (see Figs. S3–S6 in the Supplementary Information). From a theoretical point of view, we expect the endemic state (*SSV*) to be the same regardless of the size of the initial seed, at least for random networks, something which indeed holds (compare the third columns and second rows of Fig. 3 and Fig. S3). For some other network structures this might not be true. For example, for the case of $r_p$ = 0.01, $k=4$ and $\lambda$ = 0.06 there is an endemic state with positive diffusion for the 10% initial seed, whereas there is no diffusion for the 1% seed (compare first columns and second rows of Fig. 3 and Fig. S3). Nevertheless, these misalignments with respect to the endemic state are the exception, since the endemic state, in general, coincides in both settings. The most significant difference between the two situations is the erratic behaviour of the total variance (*WACR*) for relatively high values of *k* and $\lambda$ (see the third row of Fig. S3 and the third row, first column of Fig. S4). This finding is quite puzzling and not well understood. One possible explanation is that in a small seed case the sensibility to the particular location of the initial seed generates such distinct results for quite similar settings. We also note that the difference is mostly driven by changes in the long-run variance which plays a role only for the overall variance (*WACR*). This might be because, depending on whether or not the cyclical behavior of the curves in the long-run are synchronized, the total variance might change drastically from low to high values. It seems that in the 1% seed case the time each curve in the sample reaches the endemic state might vary more than in the 10% case, which, in turn, leads to more asynchronous behavior in some cases and less in others. We find that, alike in the 10% case, the network structure plays little or no role on the measures studied.

#### The susceptible-infected-recovered model

The Susceptible-Infected-Recovered (SIR) model differs from the SIS model in that infected individuals do not enter the susceptible state again but, instead, with a certain probability they recover and are immunized from then on. That is, recovery confers lasting resistance and once you recover this is an absorbing state. This process converges always to a situation in which the whole population is non-infected (either recovered or susceptible). The focus here is to analyze the diffusion peak and the time it takes to reach such peak, considering again the point-wise median as the reference curve, given a sample of simulated infected proportion curves from different parameter/model settings. In this case, the short-run variance measures the variance of the sample curves only until the diffusion peak is attained, using the same depth-based central region definition (*BWACR*) as with the SIS model. The overall (or total) variance, however, evaluates the variance throughout the whole time range (*WACR*). Preliminary results from this model suggest that there are some important differences with the SIS model. For instance, structured networks (i.e. the lattice or even small-world networks) would lead to little diffusion when the network has low density, and this is is true even for large contagion rates. The reason is that as infected individuals recover they create wholes in the network and disconnect it as the disease can no longer go through these nodes. This effect is stronger in structured networks than in random ones, in particular, if the network has low density. Also notice that the total variance is always lower than the short-run variance, a consequence of the fact that in the steady state there is indeed no variance at all since all curves reach the zero-diffusion state (see Figs. S7 and S8). Additional description about the settings of these simulations and the results are included in the Supplementary Information.

## Discussion

In this paper we study the unpredictability of a contagion process by means of a functional data-based analysis and simulations. The classical epidemiological literature has concentrated on determining under which conditions there is positive prevalence of contagion in the long-run state for random networks^13^ and more recently, also for small-world networks^30^. Pair approximations have been applied to refine mean-field dynamics in the context of structured networks^31, 32^. There are two main directions in which our paper contributes to this literature. First, by considering the whole infection curve we study the process in the short-run and not just in the long-run. We believe that focusing on the complete time course of the contagion process is important since early interventions on a potential epidemics are not only critical for preventing it, but they also might significantly affect the network structure and the contagiousness of the disease (e.g., through confinement policies and the enforcement of masks use). Therefore, the “theoretical” long-run might never be actually reached for the initial set of parameters. Second, unlike standard approaches in the literature, we analyze the properties of multiple realizations of the contagion process which allows us to deduce how much relevant information is missing when focusing on the “representative” average behavior. There is some recent interest in extending standard epidemiological models to account for stochastic outcomes and variance but this is mostly developed for random networks^8–10^. We contribute to this literature by providing an alternative and flexible approach based on simulations and functional data analysis that can easily be applied to any contagion process.

Our analysis of the SIS model on small-world networks has led to novel results. First, we have observed some degree of complementarity between the contagion rate and network density. In other words, intermediate values of the contagion rate and density lead to more diffusion than unbalanced situations (i.e., high contagion rates but low density, or viceversa). Second, given the density of the network, the variance (or unpredictability) of the process increases rapidly with the contagion rate, reaches its maximum value at the epidemic threshold and then decreases at a lower pace. Also, although the structure of the network (structured versus random) does not seem to play a strong role on any of the studied variables, we do find some regularities. For instance, in the positive diffusion regimes it is always the case that the convergence time is highest in the lattice and decreases as the randomness of the network increases. In the zero-diffusion regime the opposite holds. A similar finding occurs for the overall variance, which relies strongly on the time of convergence to the steady state, but not for the short-run variance that is even less sensitive to network structure.

Both the SIS and SIR models have in common that the probability of becoming infected depends exclusively on the number of infected neighbors at a certain time and not on the neighborhood’s size, i.e., susceptible agents do not influence against infection. There are other models of contagion in contexts of opinion formation and social persuasion for which the probability of becoming an adopter depends on the relative number of adopters instead. These models were originally studied in the context of global interactions^33^ and later on in random networks^34^ as well as small-world networks^35^. In addition, the network structure can be extended to account for more realistic features such as homophily^18,36^. Our study, therefore, can be considered as a starting point, and investigating more sophisticated processes would be a natural and promising way of proceeding with this line of research.

Our methodology allows for the prediction of the most common patterns of diffusion through simulations, by determining the most central or representative infection curves according to the modified band depth definition, where the deepest curve would be considered as the median. Moreover, it allows ranking the sample of generated curves from center-outward and defining non-parametric and robust measures of variability or uncertainty. From an empirical perspective, other applications of this functional data approach could be considered. For instance, there is an extensive literature on robust classification depth-based methods for functional data^37–39^ that could be used to infer unidentified information of the contagion process. For example, assume that the contagion rate ($\lambda$) of a new infectious disease (or of a new product) is unknown, although there is information on the network structure and the contagion curve (number of cases over time) is observed. The most plausible contagion rate (among a set of potential ones) can be inferred by calculating the model that provides a sample of curves for which the observed one is deepest and, therefore, more likely to come from. A similar argument would apply to infer the properties of the network structure through which the infectious disease is spreading given the observed infection curves. We believe that the functional approach described here has potential for various fruitful applications by combining empirical findings with simulation studies, that will allow us to better understand the properties of contagion processes.

### Supplementary Information

Supplementary Information.

## Data Availability

All data generated or analysed during this study are included in this published article and its supplementary information files.
