# Supplementary material for: Uncertainty analysis of contagion processes based on a functional approach
Source: Sci Rep. 2023 Sep 19;13:15522. doi: 10.1038/s41598-023-42041-0 (PMC10509249; doi:10.1038/s41598-023-42041-0)
Supplement: Supplementary file 1 — Supplementary Information. [file 41598_2023_42041_MOESM1_ESM.pdf]

Supplementary Information: Uncertainty analysis of contagion processes based on a functional approach

Dunia López-Pintado (Corresponding author. Universidad Pablo de Olavide, Sevilla, Spain. E-mail: dlopez@upo.es), Sara López-Pintado (Corresponding author. Northeastern University, Boston, USA. E-mail: s.lopez-pintado@northeastern.edu), Iván García-Milán (Universidad de Loyola, Sevilla, Spain. E-mail: igmilan@uloyola.es), and Zonghui Yao (Northeastern University, Boston, USA. E-mail: yao.zo@northeastern.edu)

August 30, 2023

Computation of SSP by numerical difference approximation

The calculation of the SSP requires taking the first derivative of the point-wise median curve (numerical difference approximation) and smoothing it by considering a moving average approach of a certain window (in this example, a window of size 10), as illustrated in Fig. S1. The SSP is defined as the point in time after which such estimated derivative function is close to zero, e.g., reaches a value lower than a small threshold. The threshold is computed endogenously as the average value of the smoothed derivative for the 20% final periods.

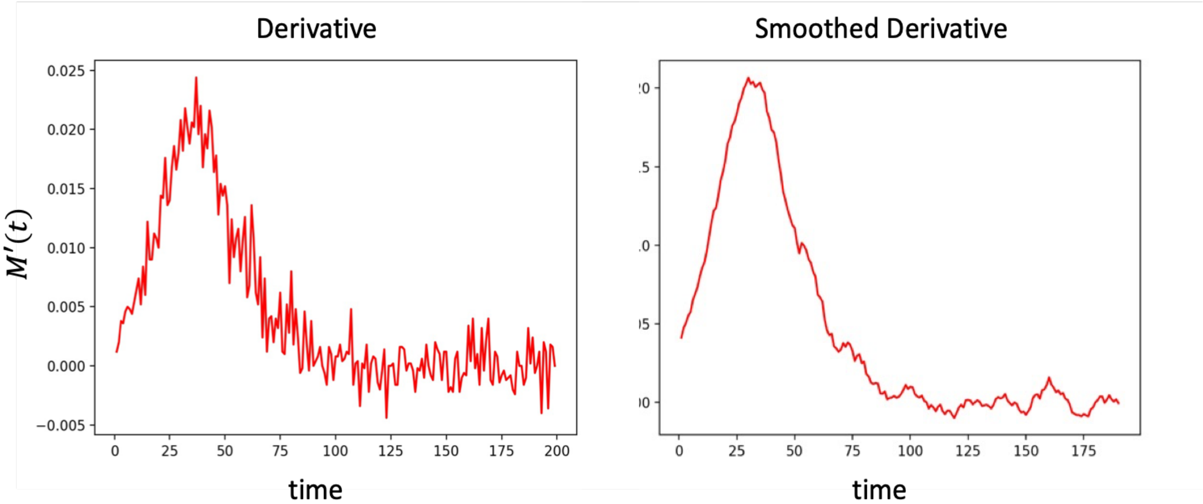

Figure S1. Computation of SSP by numerical difference approximation. Left. Derivative of (point-wise) median curve. Right. Moving average with length 10 sliding window.

Non-monotonicity of $WACR_q$

As shown in Proposition 1, $WACR_q \leq \text{WACR}_1$ for any $q \in \{1, 2, ..., n\}$. This might suggest that $WACR_q$ is decreasing with respect to q. We show by means of a counterexample, illustrated in Fig. S2, that this is not the case.

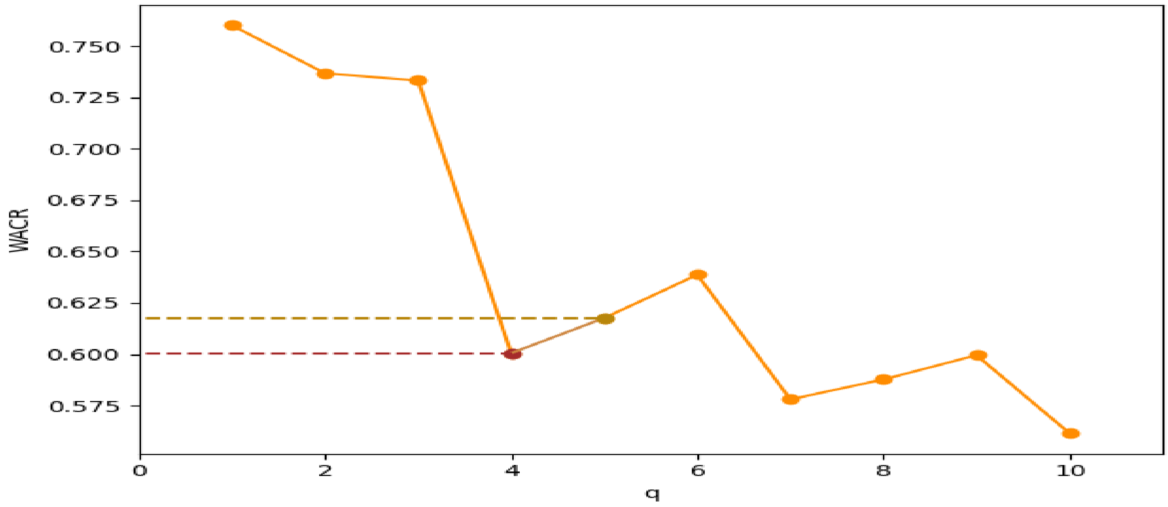

Figure S2. Non-monotonicity of $WACR_q$. Representation of $WACR_q$ for $q \in \{1,2,...,10\}$ given a network with $S = 100$ nodes, 1% clustered initial seed, k=8, r_p=0.01, λ=0.3, and a sample of $n = 100$ infected proportion curves.

Results for 1% concentrated seed

In this section we perform the same analysis as in the main text but considering a 1% concentrated initial seed. As observed in Figs. S3 and S6 the main features remain. In particular, maximum variance is attained at the epidemic threshold. Observe that if $k = 4$ and r_p=0.01 the epidemic threshold for λ is slightly higher here than in the 10% concentrated but this is the exception, as typically the endemic state coincides in both scenarios. The most important difference is the erratic behaviour of the $WACR$ for relatively large values of k and λ (see the graphs in the third row of Fig. S3 and the graph in the third row, first column of Fig. S4). Thus, the results are more sensible to the precise location of the initial seed, which can differ for different parameter configurations. The case of random 1% and 10% initial seeds provide qualitatively the same results as the 1% and 10% clustered initial seeds, respectively. These results are available upon request.

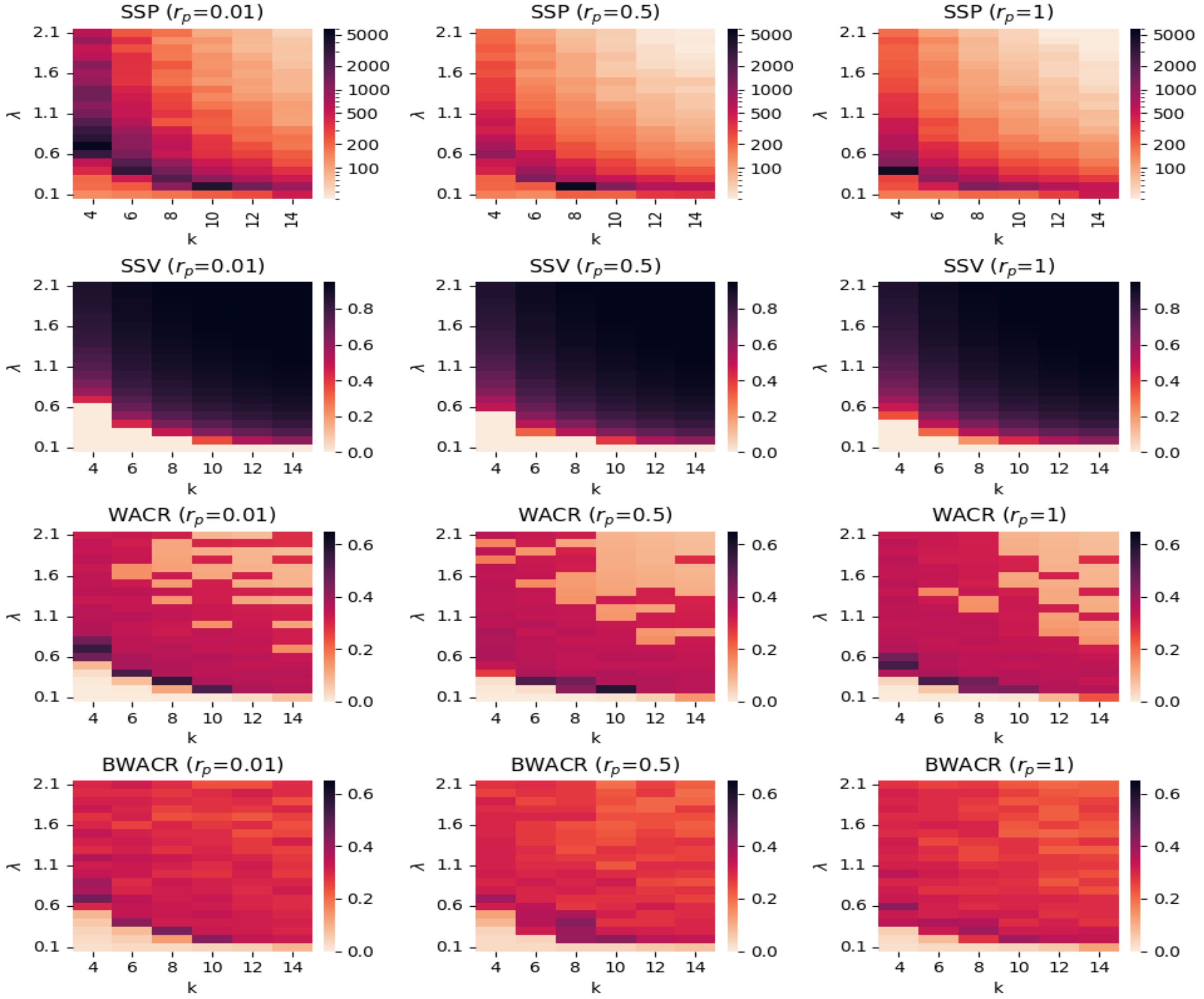

Figure S3. 1% seed case. Heatmaps of the steady state and unpredictability. *SSP*, *SSV*, *WACR* and *BWACR* values (top, middle and bottom rows, respectively) as a function of λ and k at three levels of r_p (left, middle and right columns, respectively). The darker the colour the higher the values. The *SSP* values are represented in logarithmic scale.

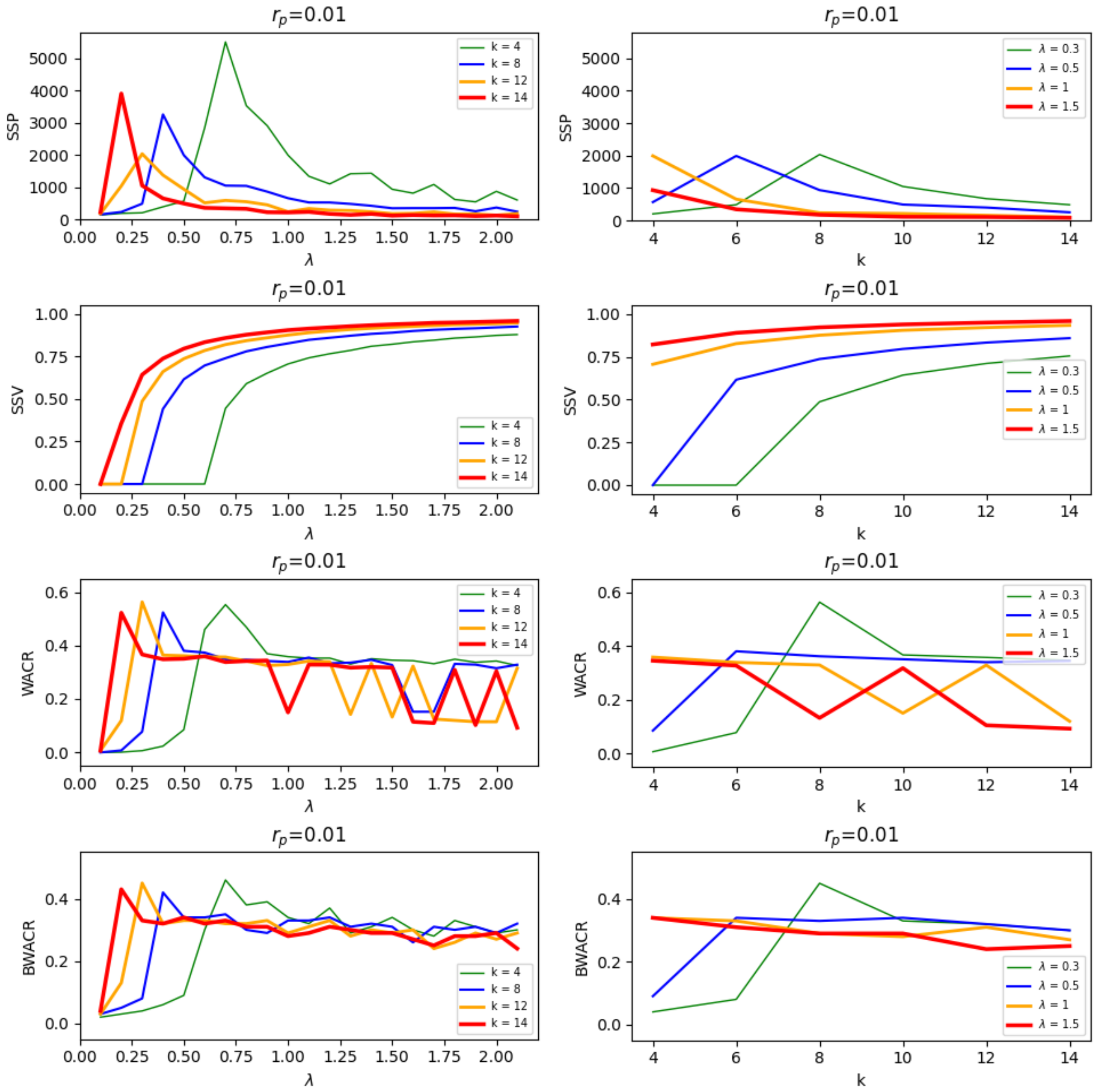

Figure S4. 1% seed case. Left column. Uncertainty and contagion rate. Representation of *SSP*, *SSV*, *WACR* and *BWACR* as a function of λ for k=4, 8, 12, and 14, and r_p=0.01. Right column. Uncertainty and network density. Representation of *SSP*, *SSV*, *WACR* and *BWACR* as a function of k for λ=0.3, 0.5, 1, and 1.5, and r_p=0.01.

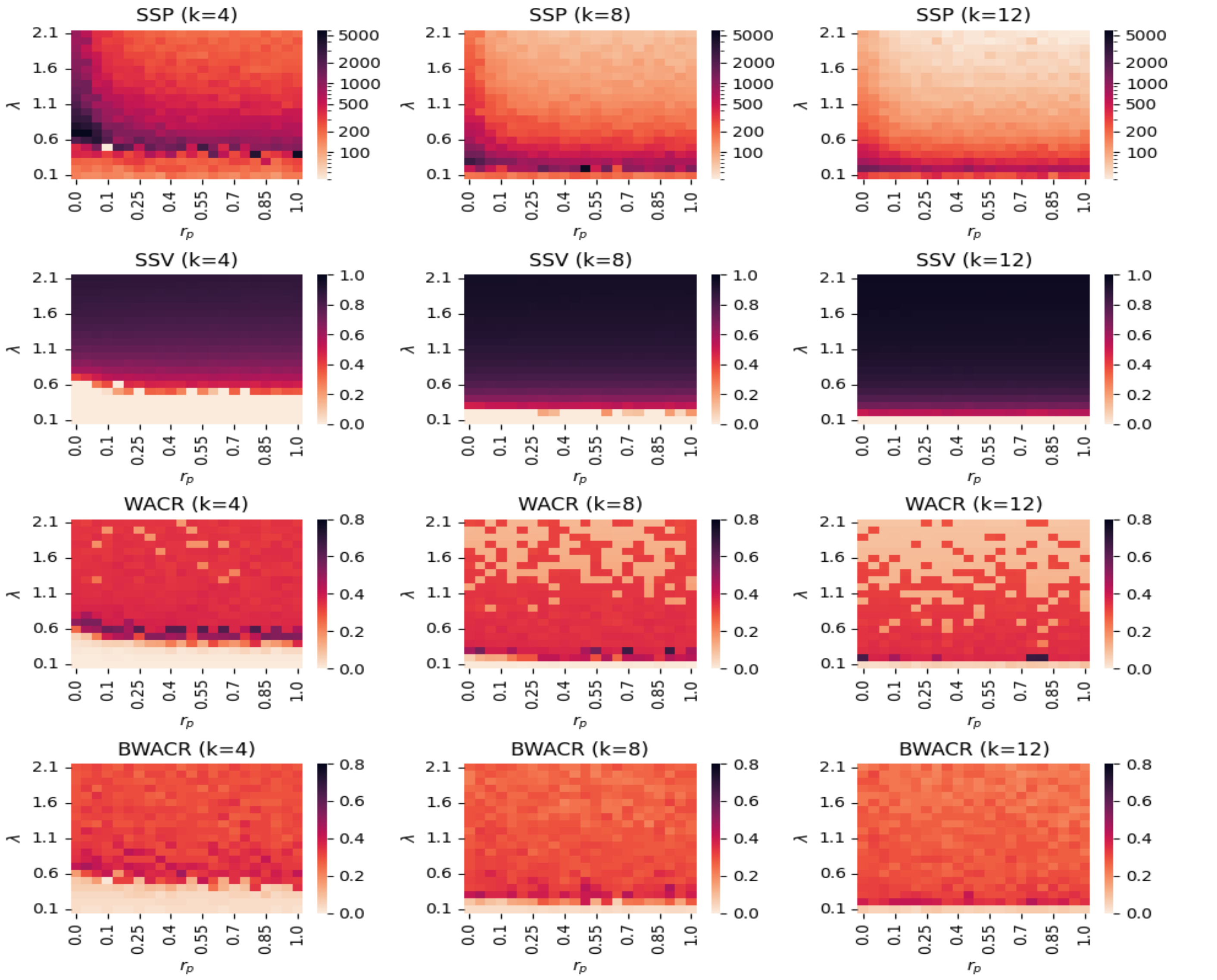

Figure S5. 1% seed case. Heatmaps of the steady state and unpredictability. *SSP*, *SSV*, *WACR* and *BWACR* values (top, middle and bottom rows, respectively) as a function of λ and r_p at three levels of k (left, middle and right columns, respectively). The darker the colour the higher the values. The *SSP* values are represented in logarithmic scale.

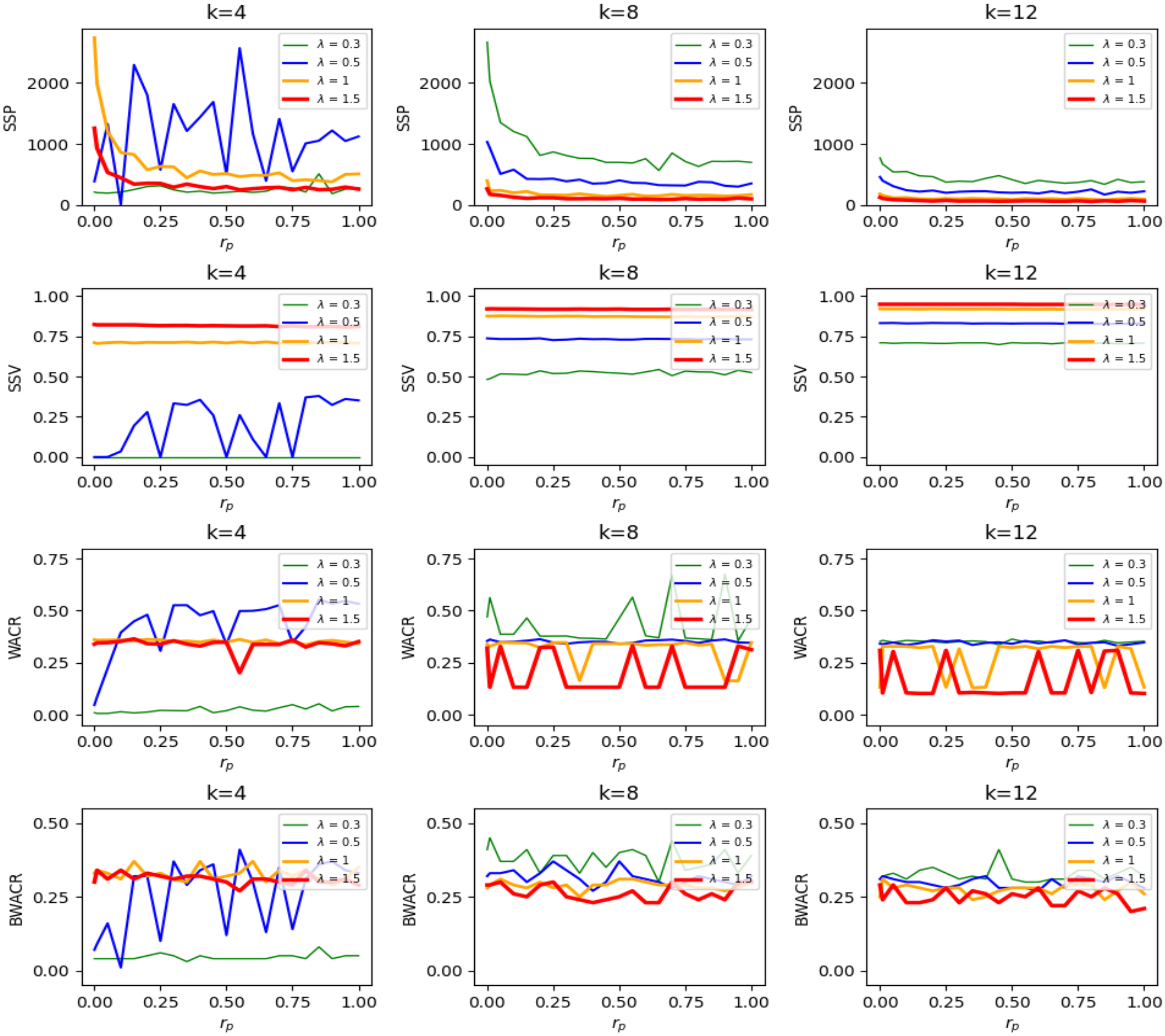

Figure S6. 1% seed case. Uncertainty and network randomness. SSP, SSV, WACR and BWACR values as a function of r_p given four values of λ and k=4, 8 and 12 (left, middle and right columns, respectively).

The SIR model

The Susceptible-Infected-Recovered (SIR) model differs from the SIS model in that infected individuals do not enter the susceptible state again but, instead, with a certain probability, they recover and are immunized from then on. In the SIR model, agents are in three possible states (susceptible, infected or recovered). A susceptible agent becomes infected with a probability β when interacting with an infectious agent. With a probability μ an infected agent becomes recovered (a state which can also be interpreted as removed). The recovered state is absorbing. For simplicity, we will assume a fixed value of μ and vary β. The key parameter is considered as $\lambda = \frac{\beta}{\mu}$. As for the SIS model, we concentrate on the infected proportion curves and compute its point-wise median. The steady state is characterized by zero diffusion and, thus, the focus of the SIR model is on the diffusion

peak. In particular, the "Maximum Point" (denoted as MP or t^*) is the first time period where $M(t)$ reaches its maximum value for $t \in [0, T]$, where T is set high enough so that the zero-diffusion state has been reached. Moreover, the "Maximum Value" (denoted as MV or x^*) is precisely $M(t^*)$. Regarding the variance measures, we implement the same concepts described in the main text of the paper. In particular, the *WACR* corresponds with the overall variance of the simulated infection proportion curves, whereas the *BWACR* measure corresponds with the variance of the simulated infection proportion sample of curves up to the diffusion peak (both based on the *MBD* central regions as before). The family of small-world networks considered for the simulations performed here are formed by $S = 100$ nodes, and a 1% seed of initially infected agents. The simulation study assumes average degrees k, ranging from 4 to 14 (only pair values), rewiring probabilities r_p, ranging from 0 to 1 and contagion rates λ taking values from 0.1 to 2.1. For every network created with parameters (k, r_p), a contagion rate λ, and a fixed initial seed, we run 100 repetitions of the SIR dynamics and derive a sample of infected proportion curves. We summarize and visualize the results in a schematic way by representing, under the different parameter specifications considered, the MP, MV, *WACR* and *BWACR* below.

In the SIR model the epidemic threshold with respect to λ can be considered as the value of λ for which there is an abrupt increase in the MV value, given k and r_p (the epidemic threshold with respect to k and r_p can be defined analogously). We observe from the second row in Fig. S7 that there generally exists an epidemic threshold with respect to λ (and k). Also, both MP and *WACR* are roughly maximized at the epidemic threshold (see first and third rows of Fig. S7). However, unlike in the SIS case, *BWACR* has an increasing trend with respect to λ (and k). Finally, as shown in Fig. S8, the effect of network randomness (i.e., r_p) is relevant in the SIR model. There typically exists an epidemic threshold with respect to r_p (e.g., the infection does not spread unless the network is sufficiently random for the case $k = 4$). Here, again, MP and *WACR* are roughly maximized at the epidemic threshold (see first and third rows in Fig. S8), whereas the result is less obvious for the *BWACR* variance.

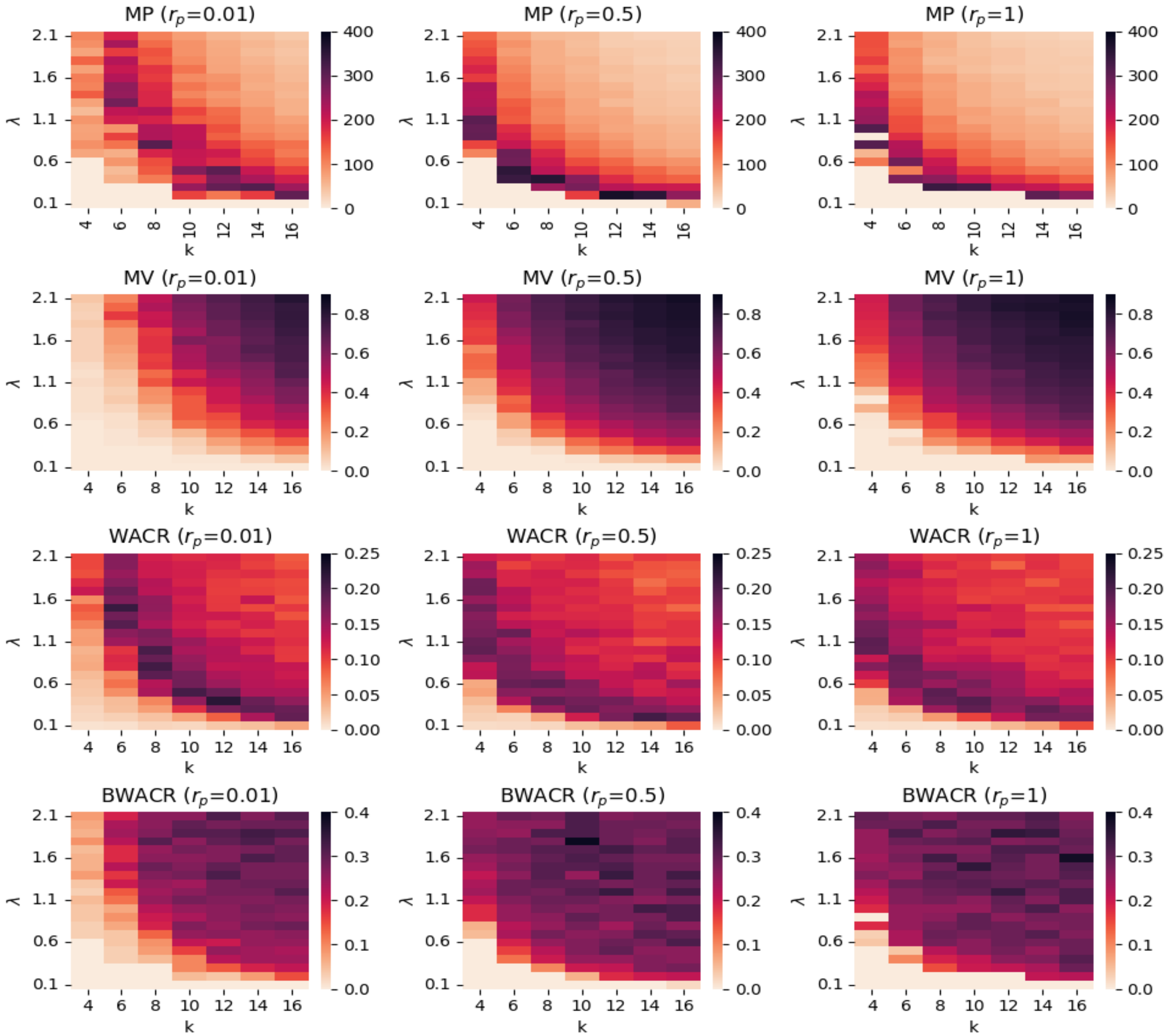

Figure S7. The SIR model. Heatmaps of the steady state and unpredictability. *SSP*, *SSV*, *WACR* and *BWACR* values (top, middle and bottom rows, respectively) as a function of λ and k at three levels of r_p (left, middle and right columns, respectively). The darker the colour the higher the values.

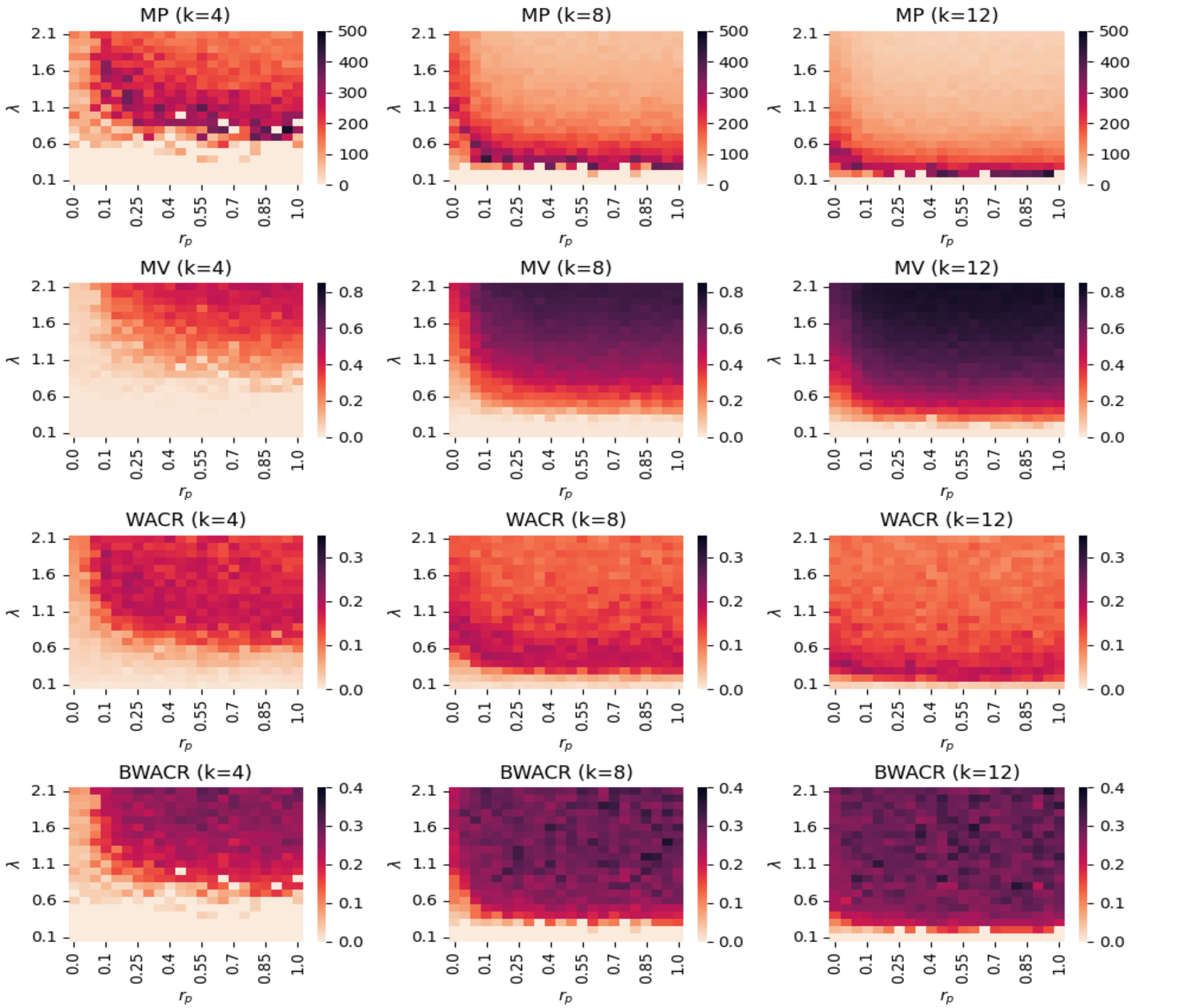

Figure S8. The SIR model. Heatmaps of the steady state and unpredictability. *SSP*, *SSV*, *WACR* and *BWACR* values (top, middle and bottom rows, respectively) as a function of λ and r_p at three levels of k (left, middle and right columns, respectively). The darker the colour the higher the values.
